# 创伤失血性休克致凝血功能异常1例

**DOI:** 10.3760/cma.j.cn121090-20241127-00486

**Published:** 2024-12

**Authors:** 宁 骆, 寅 李

**Affiliations:** 天津市第一中心医院，天津 300192 Tianjin First Central Hospital, Tianjin 300192, China

患者，男，50岁。既往体健，本次主因“左下腹、左腿砸伤1 h”就诊。入院前1 h，患者在工地干活时不慎被钢管砸伤左侧下腹部及左腿，至急诊时患者神志尚清，肢端凉，体温36.3 °C，心率120次/min，呼吸频率21次/min，血压83/52 mmHg（1 mmHg=0.133 kPa），左侧腹股沟区可见一逐渐增大的血肿，伴有左下肢肿胀。急诊予止血、抗休克、输血等对症支持治疗，完善左侧股骨计算机X线摄影提示左股骨干中下段骨折，血管造影显示左侧股动脉断裂。血常规：HGB 107 g/L, PLT 274×10^9^/L；生化常规：血肌酐（Cr）82.3 µmol/L，ALT 37.2 U/L；凝血功能：PT 14 s，APTT 39.7 s，纤维蛋白原（FIB）3.72 g/L，D-二聚体（D-dimer）6 285 µg/L；血气分析：pH=7.40，PaCO_2_ 37 mmHg，PaO_2_ 128 mmHg，HCO_3_^-^ 22.6 mmol/L，碱剩余（BE）0.5 mmol/L，血氧饱和度（SO_2_）98％，乳酸（Lac）1.8 mmol/L。急诊初步诊断：①左下腹、左下肢外伤：左侧股动脉断裂、左股骨干骨折、左小腿碾压伤伴骨筋膜室综合征；②创伤失血性休克（损失严重度评分：16分；休克指数：1.44）。急诊予限制性液体复苏，生理盐水500 ml，血凝酶2 IU（入院后30 min内）；紧急备血。行急诊手术：①左侧髂动脉、股动脉人工血管重建术；②左股骨干骨折切开复位+支架外固定术；③左小腿骨筋膜室切开减张术。术后转入复苏室（手术时间约3.5～4 h，术中去甲肾上腺素0.1～0.15 µg·kg^−1^·min^−1^，维持收缩压90～110 mmHg）。入院8 h内共输注悬浮红细胞8 IU，新鲜冰冻血浆800 ml，人纤维蛋白原5 g。患者HGB由67 g/L回升到80 g/L，PT由22.8 s缩短到16.7 s，APPT由65.5 s缩短到49.1 s，FIB由1.32 g/L回升到1.67 g/L，患者PLT从入院时的274×10^9^/L降至73×10^9^/L。患者在复苏室神志不清，自主呼吸微弱，血压低，予去甲肾上腺素泵入升压，尿量0～15 ml/h，血肌酐进行性升高；左小腿皮温低、血运差、肿胀，故转入ICU。

转入时体温36.5 °C，心率98次/min，呼吸频率21次/min，血压91/54 mmHg（去甲肾上腺素0.1 µg·kg^−1^·min^−1^），麻醉状态，保留气管插管接呼吸机，心肺（−），腹部平坦，无肌紧张，肝脾肋下未及，右侧巴氏征阴性。专科情况：左下腹腹股沟区敷料加压包扎，左小腿敷料包扎，局部敷料可见血性渗出较多，左大腿可见外固定架，左小腿血运差，皮温低，足背动脉搏动触摸不清，左足趾、踝关节、膝关节被动活动僵硬受限。复查血常规：WBC 8.66×10^9^/L，HGB 88 g/L，PLT 63×10^9^/L，中性粒细胞百分比70.9％；生化常规：K^+^ 5.3 mmol/L，白蛋白（ALB）24.7g/L，尿素氮（BUN）9.92 mmol/L，Cr 140 µmol/L，ALT 109.9 U/L，AST 384.7 U/L，肌酸激酶（CK）12 823 U/L，肌红蛋白（MYO）>3 909 ng/ml；血气分析：pH=7.32，PaCO_2_ 38 mmHg，PaO_2_ 127 mmHg，HCO_3_^-^ 17.1 mmol/L，BE −6.5 mmol/L，SO_2_ 98％，Lac 1.5 mmol/L；凝血功能：PT 14.1 s，APTT 39.7 s，FIB 2.11 g/L，D-dimer>10 000 µg/L。转入ICU时诊断：①创伤失血性休克；②左下腹、左下肢外伤：左侧股动脉断裂；左股静脉挫伤；左股神经挫伤；左侧股骨干粉碎性骨折；左小腿碾压伤伴骨筋膜室综合征；③急性肾损伤；④代谢性酸中毒；⑤血小板减少症；⑥凝血功能异常；⑦电解质代谢紊乱；⑧肝功能损伤；⑨低蛋白血症（APACHEⅡ评分28分，SOFA评分10分）；转入ICU当天，于床旁再次行左小腿切开减张术；术后查左下肢DSA：左侧腘动脉、胫前动脉及胫后动脉血栓形成；血管外科建议取栓、保肢治疗，家属拒绝。ICU予抗休克、抗感染、血液净化、镇痛镇静、营养支持等综合治疗。入院第3天，患者血流动力学趋于稳定，停用升压药；间断输血、输注新鲜冰冻血浆，输注1个治疗量血小板。入院第8天，行左下肢伤口清创术：术中见左小腿胫前部分皮肤坏死，左大腿股内侧肌大部分坏死；左小腿腓肠肌近端电刀刺激肌纤维可见收缩，余腓骨长短肌、趾长伸肌、胫前肌、比目鱼肌、趾长屈肌均无活力，肌肉色灰白，术中行原减张伤口内清创，切除上述部分坏死肌肉，左足血运尚可，大量过氧化氢水溶液、生理盐水、安尔碘皮肤消毒剂再次冲洗，伤口内留置抗生素骨水泥。此后患者尿量增多，但肌酐下降不明显，仍需血液净化治疗。考虑患者肾功能恢复不理想与下肢病变有关，与家属充分沟通后，同意截肢。入院第15天，骨科行左侧膝关节离断，伤口清创+负压封闭引流术。术后患者尿量进一步增多，肌酐逐步下降，停止血液净化治疗。入院第22天转至骨科，转出时患者HGB 89 g/L，Cr 142 µmol/L，ALB 35 g/L。入院第29天行左下肢清创。入院第56天患者出院，出院时伤口愈合较好，HGB 96 g/L，Cr 63 µmol/L。

讨论：患肢术后短时间即出现动脉栓塞，分析其可能原因如下：①病理生理上，创伤导致的血管壁损伤、血流改变、血液成分改变均可能导致动脉血栓形成；②医源性因素，手术方式的选择可能对患者造成影响。对于无脑损伤的患者，在大出血彻底控制之前，实施“允许性低血压”的治疗策略，将收缩压维持在80～90 mmHg即可，避免大量输液。大量补液会导致血凝块移位、血液内凝血因子稀释，从而导致止血困难和出血风险增加，增加病死率。从手术角度来说，对于合并重度失血性休克、有持续出血和凝血功能障碍表现的严重创伤患者，以及难以处理的解剖损伤、操作耗时等情况，推荐实施损伤控制性手术，使患者获得复苏时间，有机会再进行完整、合理的再次或分期手术。如果此时再进行确定性手术，复杂的外科手术及麻醉会进一步引起失血和热量丢失。酸中毒、全身炎症反应综合征和免疫系统损害使患者自身的创伤修复能力严重受损，导致术后出现严重并发症。该患者术后短时间内出现动脉血栓形成，与其临床结局密切相关。纵观其治疗过程，早期实施限制性液体复苏对患者有益。但确定性手术延长了手术时间，对患者病理生理状态有一定影响。实施临时转流等损伤控制性手术可能使患者预后更佳。提示临床医师，针对严重创伤患者，应尽快排除创伤性凝血病，选择合适的手术方式，并动态评估患者的凝血功能，合理使用止血药物，及时调整治疗方案。

